# Effect of Lactose-Reduction in Murciano-Granadina Semi-Hard Goat Cheese on Physicochemical and Sensory Characteristics

**DOI:** 10.3390/foods12050996

**Published:** 2023-02-26

**Authors:** Ángel Luis López Ruiz, Francisco de Asís Ruiz Morales, Pilar Ruiz Pérez-Cacho, Hortensia Galán-Soldevilla

**Affiliations:** 1 Departamento de Bromatología y Tecnología de los Alimentos, Universidad de Córdoba, Campus de Rabanales, 14071 Córdoba, Spain; 2 Centro IFAPA Hinojosa del Duque, 14270 Córdoba, Spain; 3 Centro IFAPA Camino de Purchil, 18004 Granada, Spain

**Keywords:** dairy product, lactose-free product, physicochemical composition, sensory profile

## Abstract

Semi-hard pressed goat’s cheese, a traditional matured cheese in Andalusia, has a residual lactose content that may affect people with intolerance to that carbohydrate. Nowadays, lactose-free dairy products are characterized by presenting a scant sensory quality, far removed from their traditional profile for their pronounced sweet and bitter taste and aroma related to Maillard reactions. The aim of this work was to make a cheese with a similar sensory profile to that of the traditional Andalusian one but without lactose. For this purpose, the doses of the enzyme lactase that would be necessary to add to the milk were investigated so that, during the manufacturing of the cheese, there would remain enough lactose for the starter cultures to trigger lactic fermentation and, in turn, to spark the cheese’s own maturity processes. The results show that the combined action of lactase (0.125 g/L, 0.250 g/L, 0.5 g/L, and 1 g/L) and of the lactic bacteria reduces the final content of lactose to below 0.01%, complying with the European Authority of Food Safety’s recommendations for considering the cheeses as being under the denomination “lactose-free”. The physicochemical and sensory values resulting from the different batches of cheese obtained indicate that the lowest dose studied (0.125 g/L) had very similar ones to those of the control cheese.

## 1. Introduction

Most of the world’s adult population is intolerant to lactose, with some authors having reported as much as 70%, although there is great geographical variability. In northern, southern, and western Europe, the lactose malabsorption prevalence was 28% [1]. Lactose intolerance occurs when the body is unable to produce the necessary amount of lactase and, consequently, undigested dietary lactose enters the large intestine, where it acts as a fermentable substrate for the colonic microflora and causes osmotic diarrhea and other symptoms [2].

The food industry has recently developed a wide range of products for people with this type of intolerance. However, rules for low-lactose foods are currently not harmonized in the European Union. In either case, the Spanish Agency for Food Safety and Nutrition (AESAN) has established that a food product labeled as “lactose-free” should have less than 0.01% lactose and 1% for low-lactose products [3]. In this context, the dairy industry is responding to consumer demands by offering a wide range of low-lactose or lactose-free dairy products.

Lactose can be removed from dairy products by hydrolyzing it in the milk by means of the β galactosidase enzyme, which converts lactose into glucose and galactose, or with the prior ultrafiltration of the milk, followed by the action of the lactase enzyme [4,5]. The hydrolysis of lactose can influence both the technological and the sensory properties of products [6]. Considerable efforts have been dedicated to studying low/lactose-free milk in recent years [7,8,9,10,11,12], whereas the characterization of other lactose-free dairy products has received very little attention. In fact, cheeses have been very little studied [6,13,14], and as far as we know, there are no studies on lactose-free or lactose-reduced goat cheeses. Lactose-free dairy products are characterized by their pronounced sweet and bitter taste and aroma related to Maillard reactions [9,10,14,15].

Andalusia has a large variety of excellent quality cheeses linked to the territory of origin, and they are made in small, artisanal, cheese factories with goat milk from Andalusia’s indigenous breeds (Malagueña, Murciano-Granadina, Florida, Payoya), this being the main Spanish goat-milk-producing region, with 45% of the national production. These kinds of cheeses are artisanal products made of raw/pasteurized milk, usually curded with animal rennet, pressed, salted, and ripened over different periods of time, depending on the final product manufactured [16]. The Andalusian artisanal dairy industry is interested in expanding its market with innovative new products, among which are lactose-free ones, which could be attractive to a considerable number of consumers demanding healthy products. That is why it is important to understand the sensory characteristics of lactose-reduced or lactose-free goat cheese as compared to traditional goat cheese.

In this context, this work has proposed as its main aim to evaluate the effect of lactose-reduction on the physicochemical and sensory characteristics of Murciano-Granadina semi-hard goat cheese to obtain a lactose-free cheese with a similar sensory profile to traditional ones.

## 2. Materials and Methods

### 2.1. Lactose Removal Process

The commercially available enzyme GODO YNL2 (Danisco, France) produced by *Kluyveromyces lactis* was used in the present study. The conditions of the enzymatic hydrolysis of lactose in goat’s milk were preliminarily assessed on a laboratory scale. Thus, in order to select the most suitable dose of lactase for making these cheeses, the amount of residual lactose remaining in the milk after the addition of lactase and in the cheese after its manufacture without adding starter cultures was investigated using the method described in Section 2.3. The starting-out raw milk had an initial lactose content of 4.75 g/100 mL milk, and after the addition of different lactase doses (0.25, 0.5, 1, and 2 g/L milk) and incubation for 1 h, the lactose content was reduced in the milk to 2.31, 1.26, 0.33, and 0.05 g/100 mL and to 0.47, 0.08, 0.02, and 0.01 g/100 g in the cheese, respectively. The control cheese maintained a residual amount of lactose of 2.37 g/100 g cheese.

### 2.2. Cheese Processing

Cheeses were produced in the Pilot Plant of the Agricultural Research Training Centre in Hinojosa del Duque (Cordoba, Spain) by a traditional manufacturing method as described by de la Haba et al. [16]. The experiment was conducted using Murciano-Granadina whole pasteurized goat milk (72 °C/20 s). The milk was cooled to 31 °C, and the starter cultures (Choozit MA 4001, Danisco, France), together with the commercial lactase enzyme (GODO YNL2, Danisco, France) at the dosage selected in the preliminary study (0.125, 0.25, 0.50, and 1.0 g/L), were added directly to the milk and incubated for 60 min. It used freeze-dried concentrated lactic starter cultures (*Lactococcus lactis* subsp. *lactis*, *Lactococcus lactis* subsp. *cremoris, Lactococcus lactis* subsp. *lactis biovar. Diacetylactis*, and *Streptococcus thermophilus*). To carry out the dosage, a dilution of the starter with UHT milk was made in glass bottles sterilized in an autoclave. Then, calcium chloride (0.30 mL/L of milk, Laboratorios Arroyo-Spain) and kid rennet (0.32 mL/L—1:10.000, Cuajos Caporal, Spain), sufficient to coagulate the milk within 50 min, were also added. The curds were subsequently cut to obtain grains of about 4–6 mm and submitted to slow, continuous mixing to increase the temperature to 36 °C. Then, the curd was drained off and molded into pieces of 0.5 kg. Cheeses were pressed (0.5–2.0 bar) for 90 min. After reaching a pH of 5.5, they were demolded and then immersed in brine (17° Baume at 10 °C for 65 min). Finally, they were ripened in chambers for 48 h at 10 °C and 70% HR and for 45 days at 12 °C and 85% HR.

Therefore, the experimental design comprised 4 different cheese formulations made with increasing lactase doses (0.125, 0.25, 0.50, and 1.0 g lactase/L milk) and a control at three different times of the year (spring, summer, and fall). In all, 45 cheeses were made (4 doses and the control × 3 cheeses × 3 times).

### 2.3. Chemical Composition

The milk’s basic chemical composition (total solids (TS), fat, protein) was analyzed with a Milkoskan™ FT (Fourier transform infrared spectrometry) in Foss equipment (Foss Electric, Hillerød, Denmark). In addition, somatic cell count (SCC) was obtained using a Fossomatic™ 7 (Foss Electric, Hillerød, Denmark), and total bacterial counts were measured with BactoScan™ (Foss Electric, Hillerød, Denmark). Finally, lactose content was determined using liquid chromatography coupled with pulsed amperometric detection (LC-PAD) (Metrohm 930 Compact IC Flex, Herisau, Switzerland).

In the cheese, its pH, fat, total solids (TS), lactose, and sodium chloride contents were measured. The pH was analyzed with a pH meter (HANNA FHT-803) with a pH electrode. Fat content was measured according to ISO/IDF methods [17]. TS and sodium chloride contents were determined following the official method [18]. The sodium chloride content was analyzed using back titration with potassium thiocyanate to determine the concentration of chloride ions in the solution based on the Volhard method. Lactose content was also measured using liquid chromatography coupled with pulsed amperometric detection (LC-PAD) (Metrohm 930 Compact IC Flex, Switzerland). Three cheeses from each batch were analyzed. All the determinations were made in duplicate, and each pair of data was averaged.

### 2.4. Sensory Profile

The sensory profile was outlined following Ruiz Pérez-Cacho et al. [19]. The analyses were performed in the Sensory Laboratory of the Departamento de Bromatología y Tecnología de los Alimentos at the Universidad de Córdoba (Spain), which is equipped in accordance with ISO 8586:2012 [20]. The samples were prepared at least 2 h before their analysis so that they reached a temperature ranging between 16 and 18 °C. They were cut into triangular-shaped portions (0.5 cm thick, 6–7 cm length) and presented in closed, disposable Petri dishes. Each taster received two portions of cheese per sample, one to evaluate its color intensity and texture and the other its flavor. Eight highly trained panelists from the Córdoba University Sensory Laboratory collaborated in this research. The panel had previous experience in the sensory analysis of cheeses [19,21]. Testing was performed in the sensory test area under the conditions specified in the standard ISO 22935-2:2009 [22]. All the analyses were conducted in the morning. Twenty-two attributes were analyzed: 1 for appearance, 13 for flavor (4 for odor (orthonasal) perception, 4 for aroma (retronasal) perception, 4 for basic tastes and persistence), and 8 for texture. Between tastings, the assessors were able to drink mineral water to clean their taste buds.

### 2.5. Data Processing and Statistical Analysis

All the statistical tests were performed with the SPSS 17 program. A basic descriptive statistical analysis (mean and standard deviation) and a one-way ANOVA were applied for the milk’s nutritional composition. In addition, a basic descriptive statistical analysis, and a two-way ANOVA (season × doses) were carried out for each cheese’s physicochemical and sensory characteristics. To test mean differences, Tukey tests at a 95% confidence level (*p* < 0.05) were used.

## 3. Results and Discussion

### 3.1. Lactose Removal Process

As expected, the lactose concentration decreased in accordance with the increase in the lactase concentration added to the milk previously pasteurized. The addition of lactase at a concentration of 0.25 g/L reduced the lactose concentration in the milk by 50%, whereas a concentration of lactase of 2.0 g/L was needed to reduce the concentration of lactose in the cheese to below 0.01%. Therefore, the highest dose of lactase in the milk was rejected (2 g/L milk), as it was considered that too little lactose would remain available for the ferments to act during the cheese’s maturation, and that the characteristic aromas of this type of cheese would not develop. Thus, a lower dose than those assayed was selected (0.125 g/L milk), as it was believed that the amount of residual lactose in the milk would be sufficient to produce the cheese’s aromas during its fermentation and leave a final lactose value in the cheese beneath the one permitted in the legislation, i.e., <0.01% [3]. Thus, for this study, the doses 0.125, 0.25, 0.50, and 1.0 g lactase/L of milk were established.

### 3.2. Chemical Composition

#### 3.2.1. Milk Chemical Composition

Table 1 reports mean values, standard deviation, and analysis of variance (season) for total solids (TS), fat, proteins, fat/proteins, lactose, somatic cells, and total bacterial count of the raw goat’s milk collected from spring (S1), summer (S2) and the fall (S3).

The TS, fat, protein, and lactose contents of milk ranged from 12.7 to 13.8; 4.08 to 4.84; 3.31 to 3.51; and 4.59 to 4.75 g/100 mL milk, respectively. In general, the TS, fat, and protein contents of the milk used for the manufacture of our cheeses fall within the range for Murciano-Granadina goat milk [23,24,25]. A significant change in the milk composition was observed between batches for all the parameters studied (*p* < 0.001) except SSC and TBC, with the highest values in all the parameters for milk collected in the spring and the lowest for the milk collected in the summer. The chemical composition of raw goat’s milk depends on many factors such as the management system of the goats and/or climate conditions [26,27,28]. Pino et al. [28] showed that seasonality had a significant effect on milk fat and lactose, significantly decreasing the fat percentage from January to March, whereas it was quite constant until June. The lactose content was nearly constant throughout the investigated period, although a significantly low value was detected in June. However, in Spain, in the Pedroches district (Córdoba), most Murciano-Granadina goat herds follow an intensive regime so that the changes in their milk’s gross composition are due to their reproduction management and to climate conditions. The milk is of an exceptional hygienic quality since its total bacterial count was found to be much lower than that of the legal limit established by European regulations, i.e., <500.000 Log10 cfu/mL [29].

#### 3.2.2. Cheese Chemical Composition

Table 2 shows the mean values, standard deviation, and analysis of variance (season × doses) of Murciano-Granadina semi-hard cheese’s physicochemical parameters. The pH, TS, fat, fat/TS, and NaCl content values found in our study for cheeses made from Murciano-Granadina goats’ milk were like those reported for these cheeses in an earlier study for artisanal Andalusian cheeses [16]. With regard to the pH, significant differences were observed (*p* < 0.001), both due to the variability itself in the composition of the raw milk (season) and to the lactase doses employed, which start from milk with different amounts of lactose, which influence fermentation and, therefore, the lactic acid production responsible for the regulation of the cheese’s pH [13]. Thus, the lowest lactase doses corresponded to lower pH because of higher acidification. With respect to the dry extract (TS), there were also significant differences between seasons and lactose doses due to the variability in the chemical composition of the raw milk (Table 1). Increased fat and protein contents in the milk influenced cheese yield not only for the larger amount of nutrients available, but also for the improved efficiency of the recovery in the curd of all the nutrients [30].

With respect to the content in fat and fat/TS, a lower fat content (30.0 ± 1.8 g/100 g cheese) should be noted in the cheeses made with March milk than in those made with July and October milk (31.4 ± 0.5 and 32.1 ± 0.4 g/100 g cheese, respectively). This was because, in Spain, in artisanal goat cheese manufacturers, the milk is not standardized in fatty matter. Thus, the spring milk presented a high fat/protein ratio (38 ± 0.04, Table 1), which caused a reduction in the fat retention in the cheese during its transformation [31]. So, for these parameters, only significant differences between seasons (*p* < 0.001) were observed since the milk’s delactosing process in itself did not affect the content of fat. In relation to the salt content, significant differences (*p* < 0.01) were noted between seasons and between lactose doses. This could be due to the absorption of salt during the salting stage diminishing as the pH increased [31]. Finally, as for the lactose content, all the cheeses made with lactose-reduced milk, regardless of the lactose dose added, presented a residual lactose level of under the 0.01% stipulated by the Spanish Agency of Food Safety and Nutrition for lactose-free products [3]. Therefore, the cheese’s composition (Table 2) is more affected by the milk (season) and the pH by its delactosing degree (doses).

### 3.3. Cheese Sensory Profile

Table 3 and Table 4 show mean values, standard deviation, and analysis of variance (season × doses) of Murciano-Granadina semi-hard cheese flavor attributes. The results show that lactose-free cheeses present the same qualitative flavor profile as traditional cheese (control), except for the sweet taste peculiar to lactose-free cheeses (Table 4). This could be due to the hydrolysis of lactose to glucose and galactose in the milk, which gives a sweeter taste to the milk and that can participate in Maillard reactions [7,9]. Similarly to our findings, Shakeel-Ur-Rehman et al. [13] observed an increase in a sweet taste for lactose-free cheddar cheese with respect to traditional cheddar cheese. Leite et al. [32] showed that lactose-free stuffed coalho cheese achieved the highest acceptance rates among consumers due to its sweet taste.

Regarding the quantitative flavor profile (Table 3 and Table 4), the results of the ANOVA analyses showed that the effect of the lactase dose used in the delactosing of the milk was more marked than the seasonal variability itself of the milk in most of the flavor attributes, except for yogurt odor/aroma. Ruiz Pérez-Cacho et al. [19] observed that the aroma of yogurt in artisanal Andalusian cheeses made with Murciano-Granadina goats’ milk was associated with that goat breed. The cheeses with the highest doses of lactase presented a stronger intensity of odor/aroma of butter and cake; they were sweeter, more bitter, and less acidic or salty. Some researchers suggest that lactose-free dairy products are more likely to undergo a Maillard reaction due to the presence of a larger amount of reducing sugars and an increased level of free amino acids than a product containing unhydrolyzed lactose. They also observed that the degree of proteolysis was significantly higher in the lactose-hydrolyzed products compared to the conventional dairy ones, releasing bitter-tasting peptides responsible for their bitter taste [10,12,14,15]. In the same way, other authors have highlighted the increase in sweet and bitter flavors in cheddar [13] and in mozzarella lactose-free cheeses [14]. Lastly, in our study, cheeses with the highest dose of the enzyme (1 g/L milk) were also described as having a chemical taint and a metallic aftertaste, which could be attributed to the enzyme itself.

Table 5 shows mean values, standard deviation, and analysis of variance (season x doses) of Murciano-Granadina semi-hard cheese color intensity and texture attributes. The same as in the flavor profile, the ANOVA analyses results showed that the effect of the lactase dose was more significant than the seasonal variability of the milk itself, i.e., in its color intensity and in most of its texture attributes, except chewiness. Thus, the cheeses with the highest doses of lactase were darker, elastic, soft, tender, moist, soluble, and creamy, and their grain size was smaller than that of traditional cheese (control) due to the cheese’s lower acidification (Table 2). Other authors also observed differences in texture between traditional cheeses and cheeses free from, or with reduced, lactose. Shakeel-Ur-Rehman et al. [13] described a softer texture in the lactose-reduced cheddar than in the control. Cincotta et al. [14] found a more elastic texture and moisture in lactose-free mozzarella cheese than in the traditional one. Regarding the seasonal variability of the milk (season), the cheeses made from spring milk were darker and had a more elastic, softer, more tender, and soluble texture than cheeses made from summer or fall milk. This could be related to the actual composition of the milk (Table 1), with the highest values in all the parameters (fat, protein, and lactose) for milk collected from spring batches.

**Figure 1:**
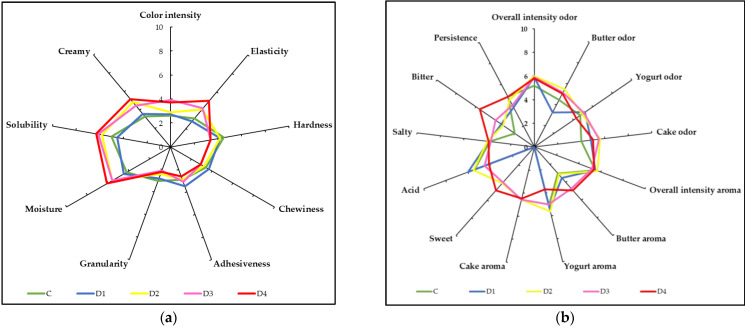
Quantitative profile of control and lactose-free Murciano-Granadina semi-hard goat cheese at different lactase doses (C = control; D1 = 0.125 g/L milk; D2 = 0.25 g/L milk; D3 = 0.5 g/L milk; D = 1.0 g/L milk). (**a**) texture profile; (**b**) Flavor profile.

The means of each lactase dose for each sensory attribute are shown as spider-web diagrams in Figure 1. The Murciano-Granadina semi-hard goat cheese sensory profile obtained in our study coincides with that observed in an earlier study for Artisanal Andalusian cheeses [19]. Thus, this artisanal cheese (Figure 1-C) is characterized by being pale in color, with olfactory notes of yogurt, butter, and cake; an acidic, salty taste; a medium persistence; and a soft, tender, and creamy texture, with low solubility. It should be noted that the sensory profiles of the cheeses made with the highest doses of lactase (Figure 1-D3 and D4) are different from those of traditional cheese, and have a profile more like that described by other authors for cheeses with reduced lactose or lactose-free, with accentuated sweet and bitter tastes and elastic, soft and moist textures [13,14]. Conversely, the sensory profile of the cheese made with the lowest dose of lactase (Figure 1-D1) was the same as that of the control cheese. Accordingly, it could be assumed that, with that dose, enough residual lactose remains in the cheese to acidify and develop the cheese’s aromas during its fermentation, and that the final lactose value in the cheese is below the one permitted in the legislation, <0.01% [3].

## 4. Conclusions

The composition of Murciano-Granadina semi-hard goat cheeses made with lactose-reduced milk is more affected by the raw milk composition and the pH by its delactosing degree (doses). All the cheeses, regardless of the lactose dose added, presented a residual lactose level of under 0.01% according to current regulations for lactose-free products. Cheeses made with the lowest dose of lactase presented the same sensory profile as traditional cheese (control), while cheeses with the highest doses of lactase were described as having accentuated sweet and bitter tastes; a chemical taint; a metallic aftertaste; and elastic, soft, and moist texture. Therefore, the results show that it is possible to make a lactose-free cheese with a similar sensory profile to that of traditional cheese. For this purpose, it is necessary to optimize the amount of lactase to be added to the milk so that sufficient lactose remains in the cheese for the typical fermentation and maturation processes to take place. This work serves as a basis for producing other types of cheeses, either lactose-free or with reduced lactose, that are similar to traditional cheeses, with milk from other species, breeds, and/or technological methods.

## Figures and Tables

**Table 1:** Mean values, standard deviation, and analysis of variance (season) of physicochemical parameters, somatic cell count and total bacterial count of raw goat’s milk.

* Season	** TS (g/100 mL)	Fat (g/100 mL)	Proteins (g/100 mL)	Fat/Proteins	Lactose (g/100 mL)	** SCC (Log_10_/mL)	** TBC (Log_10_ cfu/mL)
S1	13.8 ±0.06 ^a^	4.84 ±0.09 ^a^	3.51 ± 0.07 ^a^	1.38 ± 0.04 ^a^	4.75 ± 0.03 ^a^	6.02 ± 0.06	4.37 ± 0.22
S2	12.7 ± 0.04 ^b^	4.08 ±0.09 ^b^	3.31 ± 0.04 ^b^	1.23 ± 0.03 ^b^	4.59 ± 0.02 ^b^	6.01 ±0.04	4.28 ± 0.19
S3	13.0 ± 0.11 ^c^	4.26 ±0.18 ^c^	3.43 ± 0.09 ^c^	1.24 ± 0.03 ^b^	4.67 ± 0.05 ^c^	6.03 ± 0.04	4.48 ±0.16
F *p*	91.3 <0.001	149.8 <0.001	29.1 <0.001	82.0 <0.001	76.8 <0.001	ns	ns

Values followed by the same letter within the same column are not significantly different (*p* > 0.05) according to Tukey’s multiple range test. * S1: spring; S2; summer; S3: fall. ** TS (total solids); SCC (somatic cell count); TBC (total bacterial count).

**Table 2:** Mean values, standard deviation, and analysis of variance (season × doses) of Murciano-Granadina semi-hard cheese’s physicochemical parameters.

Parameter	^1^ Season (S)	^2^ Doses (D)	F
pH	S1: 5.16 ± 0.08 ^a^ S2: 5.17 ± 0.07 ^a^ S3: 5.11 ± 0.06 ^b^	C: 5.05 ± 0.03 ^a^ D1: 5.11 ± 0.02 ^b^ D2: 5.14 ± 0.03 ^c^ D3: 5.19 ± 0.04 ^d^ D4: 5.24 ± 0.04 ^e^	S: 51.57 *** D: 158.9 *** S × D: ns
Total Solids (TS) (g/100 g cheese)	S1: 61.0 ± 1.2 ^a^ S2: 59.7 ± 0.6 ^b^ S3: 59.9 ± 0.5 ^b^	C: 60.4 ± 0.6 ^a^ D1: 60.2 ± 0.7 ^ab^ D1: 59.9 ± 0.5 ^ab^ D3: 59.6 ± 0.6 ^b^ D4: 60.3 ± 1.6 ^a^	S: 29.5 *** D: 5.90 *** S × D: 6.17 ***
Fat (g/100 g cheese)	S1: 30.0 ± 1.8 ^a^ S2: 31.4 ± 0.5 ^b^ S3: 32.1 ± 0.4 ^b^	C: 31.1 ± 1.0 D1: 32.0 ± 1.2 D2: 30.9 ± 1.7 D3: 31.0 ± 1.4 D4: 31.4 ± 1.0	S: 16.7 *** D: ns S × D: ns
Fat/TS (g/100 g TS)	S1: 49. 1 ± 2.8 ^a^ S2: 52. 6 ± 0.9 ^b^ S3: 53. 6 ± 0.7 ^b^	C: 51.5 ± 1.9 D1: 53.1 ± 2.0 D2: 51.6 ± 3.1 D3: 52.0 ± 2.3 D4: 52.2 ± 2.6	S: 28.2 *** D: ns S × D: ns
NaCL (g/100 g cheese)	S1: 1.35 ± 0.23 ^ab^ S2: 1.41 ± 0.11 ^a^ S3: 1.30 ± 0.08 ^b^	C: 1.44 ± 0.14 ^a^ D1: 1.43 ± 0.15 ^a^ D1: 1.31 ± 0.12 ^ab^ D3: 1.31 ± 0.14 ^ab^ D4: 1.29 ± 0.14 ^b^	S: 5.70 ** D: 7.10 ** S × D: 5.20 **
Lactose (g/100 g cheese)	S1: 0.03 ± 0.04 S2: <0.01 S3: <0.01	C: 0.04 ± 0.03 D1: <0.01 D2: <0.01 D3: <0.01 D4: <0.01	

Values followed by the same letter within the same column are not significantly different (*p* > 0.05) according to Tukey’s multiple range test, ** *p* < 0.01; *** *p* < 0.001. ^1^ Season means include the values for the control cheeses, S1: spring; S2: summer; S3: fall. ^2^ Lactase doses (g/L milk): C = control; D1 = 0.125; D2 = 0.25; D3 = 0.5; D4 = 1.0.

**Table 3:** Mean values, standard deviation, and ANOVA (season × doses) of Murciano-Granadina semi-hard cheese’s odor/aroma attributes.

Attribute	^1^ Season (S)	^2^ Doses (D)	F
Overall intensity odor	S1: 5.6 ± 0.6 S2: 5.8 ± 0.4 S3: 5.9 ± 0.4	C: 5.2 ± 0.7 ^a^ D1: 5.9 ± 0.2 ^b^ D2: 6.0 ± 0.4 ^ab^ D3: 5.9 ± 0.1 ^ab^ D4: 5.8 ± 0.2 ^ab^	S: ns D: 3.53 * S × D ns
Butter odor	S1: 5.1 ± 0.5 ^a^ S2: 4.5 ± 1.4 ^b^ S3: 4.5 ± 1.4 ^b^	C: 4.5 ± 0.4 ^a^ D1: 3.3 ± 1.9 ^b^ D2: 5.5 ± 0.7 ^ac^ D3: 5.2 ± 0.4 ^ac^ D4: 5.1 ± 0.3 ^ac^	S: 5.71 * D: 21.6 *** S × D: 9.8 ***
Yogurt odor	S1: 3.9 ± 0.3 ^a^ S2: 5.2 ± 0.9 ^b^ S3: 5.5 ± 0.5 ^b^	C: 4.7 ± 1.1 D1: 5.2 ± 1.0 D2: 4.9 ± 0.8 D3: 5.1 ± 0.9 D4: 4.3 ± 0.7	S: 34.8 *** D: ns S × D: ns
Cake odor	S1: 5.3 ± 0.5 ^a^ S2: 4.9 ± 0.6 ^ab^ S3: 4.7 ± 1.0 ^b^	C: 4.0 ± 0.9 ^a^ D1: 4.8 ± 0.5 ^b^ D2: 5.6 ± 0.4 ^c^ D3: 5.5 ± 0.2 ^c^ D4: 5.0 ± 0.4 ^bc^	S: 7.42 ** D: 18.9 *** S × D: 2.84 *
Overall intensity aroma	S1: 5.0 ± 0.3 ^a^ S2: 5.6 ± 0.4 ^b^ S3: 5.7 ± 0.3 ^b^	C: 5.5 ± 0.7 ^ab^ D1: 5.5 ± 0.3 ^ab^ D2: 5.7 ± 0.5 ^a^ D3: 5.2 ± 0.2 ^b^ D4: 5.4 ± 0.2 ^ab^	S: 25.8 *** D: 3.92 * S × D: ns
Butter aroma	S1: 3.9 ± 0.8 S2: 3.8 ± 0.8 S3: 3.9 ± 1.0	C: 3.0 ± 0.1 ^a^ D1: 3.5 ± 0.5 ^b^ D2: 3.1 ± 0.1 ^ab^ D3: 4.7 ± 0.2 ^c^ D4: 4.9 ± 0.3 ^c^	S: ns D: 95.0 *** S × D: ns
Yogurt aroma	S1: 4.5 ± 1.0 ^a^ S2: 5.0 ± 0.9 ^b^ S3: 5.3 ± 0.5 ^b^	C: 5.1 ± 0.8 ^ab^ D1: 5.4 ± 0.2 ^ab^ D2: 5.6 ± 0.4 ^b^ D3: 5.0 ± 0.3 ^a^ D4: 3.7 ± 0.7 ^c^	S: 12.5 ** D: 26.4 *** S × D: ns
Cake aroma	S1: 2.8 ± 2.4 S2: 2.6 ± 2.3 S3: 2.8 ± 2.5	C: - D1: - D2: 4.6 ± 0.7 ^a^ D3: 4.6 ± 0.4 ^a^ D4: 4.5 ± 0.3 ^a^	S: ns D: 191.5 *** S × D: ns

Values followed by the same letter within the same column are not significantly different (*p* > 0.05) according to Tukey’s multiple range test, * *p* < 0.05; ** *p* < 0.01; *** *p* < 0.001. ^1^ Season means include the values for the control cheeses, S1: spring; S2: summer; S3: fall. ^2^ Lactase dose (g/L milk): C = control; D1 = 0.125; D2 = 0.25; D3 = 0.5; D4 = 1.0.

**Table 4:** Mean values, standard deviation, and ANOVA (season × doses) of Murciano-Granadina semi-hard cheese’s basic tastes and persistence.

Attribute	^1^ Season (S)	^2^ Doses (D)	F
Sweet	S1: 2.6 ± 2.3 S2: 2.5 ± 2.2 S3: 2.6 ± 2.3	C: - D1: - D2: 4.1 ± 0.5 ^a^ D3: 4.0 ± 0.1 ^a^ D4: 4.9 ± 0.4 ^b^	S: ns D: 296.0 *** S × D: ns
Acid	S1: 5.1 ± 1.1 S2: 5.1 ± 0.7 S3: 5.1 ± 0.6	C: 5.4 ± 0.4 ^a^ D1: 6.0 ± 0.3 ^a^ D2: 5.5 ± 0.4 ^a^ D3: 4.5 ± 0.1 ^b^ D4: 4.1 ± 0.5 ^b^	S: ns D: 28.6 *** S × D: ns
Salty	S1: 2.8 ± 1.0 ^a^ S2: 3.9 ± 0.9 ^b^ S3: 3.9 ± 1.0 ^b^	C: 3.8 ± 0.2 ^a^ D1: 3.9 ± 0.9 ^a^ D2: 4.0 ± 1.2 ^a^ D3: 3.7 ± 1.4 ^a^ D4: 2.2 ± 0.2 ^b^	S: 13.7 *** D: 9.96 *** S × D: 3.23 *
Bitter	S1: 3.0 ± 1.3 ^a^ S2: 3.6 ± 1.5 ^b^ S3: 3.6 ± 1.4 ^b^	C: 2.0 ± 0.2 ^a^ D1: 3.4 ± 0.3 ^b^ D2: 3.2 ± 0.5 ^b^ D3: 4.0 ± 0.9 ^c^ D4: 5.6 ± 0.2 ^d^	S: 50.2 *** D: 488.*** S × D: 13.9 ***
Persistence	S1: 4.0 ± 0.7 ^a^ S2: 4.4 ± 0.3 ^b^ S3: 4.6 ± 0.4 ^b^	C: 4.8 ± 0.2 ^a^ D1: 3.8 ± 0.6 ^b^ D2: 4.5 ± 0.5 ^a^ D3: 4.0 ± 0.2 ^b^ D4: 4.8 ± 0.3 ^a^	S: 17.3 *** D: 25.8 *** S × D: 4.23 **

Values followed by the same letter within the same column are not significantly different (*p* > 0.05) according to Tukey’s multiple range test, *p < 0.05; ** *p* < 0.01; *** *p* < 0.001. ^1^ Season means include the values for the control cheeses, S1: spring; S2: summer; S3: fall. ^2^ Lactase dose (g/L milk): C = control; D1 = 0.125; D2 = 0.25; D3 = 0.5; D4 = 1.0.

**Table 5:** Mean values, standard deviation, and ANOVA (season x doses) of Murciano-Granadina semi-hard cheese’s color intensity and texture attributes.

Attribute	^1^ Season	^2^ Doses	F
Color intensity	S1: 3.6 ± 0.9 ^a^ S2: 3.1 ± 0.4 ^b^ S3: 3.1 ± 0.4 ^b^	C: 2.6 ± 0.4 ^a^ D1: 2.7 ± 0.3 ^a^ D2: 2.9 ± 0.3 ^a^ D3: 3.9 ± 0.4 ^b^ D4: 3.7 ± 0.9 ^b^	S: 14.4 *** D: 27.1 *** S × D: 7.6 ***
Elasticity	S1: 3.2 ± 1.1 ^a^ S2: 4.0 ± 0.8 ^b^ S3: 4.3 ± 0.8 ^c^	C: 3.1 ± 0.6 ^a^ D1: 2.8 ± 0.6 ^b^ D2: 4.1 ± 1.1 ^c^ D3: 4.2 ± 0.3 ^c^ D4: 5.0 ± 0.3 ^d^	S: 89.4 *** D: 128.6 *** S × D: 8.43 ***
Hardness	S1: 3.7 ± 0.5 ^a^ S2: 4.0 ± 0.5 ^b^ S3: 4.0 ± 0.6 ^b^	C: 4.5 ± 0.3 ^a^ D1: 4.2 ± 0.3 ^a^ D2: 4.2 ± 0.3 ^a^ D3: 3.4 ± 0.2 ^b^ D4: 3.4 ± 0.2 ^b^	S: 4.77 * D: 23.1 *** S × D: ns
Chewiness	S1: 2.8 ± 0.4 ^a^ S2: 3.4 ± 0.3 ^b^ S3: 3.5 ± 0.5 ^b^	C: 3.4 ± 0.3 ^a^ D1: 3.7 ± 0.4 ^b^ D2: 3.2 ± 0.8 ^ac^ D3: 2.9 ± 0.2 ^c^ D4: 2.9 ± 0.2 ^c^	S: 60.3 *** D: 26.3 *** S × D: 6.65 **
Adhesiveness	S1: 2.9 ± 0.6 ^a^ S2: 3.2 ± 0.3 ^b^ S3: 2.9 ± 0.6 ^ab^	C: 2.9 ± 0.7 ^ab^ D1: 3.5 ± 0.3 ^c^ D2: 2.9 ± 0.5 ^ab^ D3: 3.1 ± 0.4 ^bc^ D4: 2.6 ± 0.3 ^a^	S: 4.30 * D: 8.02 ** S × D: 5.87 **
Granularity	S1: 2.2 ± 0.6 ^a^ S2: 2.7 ± 0.5 ^b^ S3: 2.7 ± 0.3 ^b^	C: 3.0 ± 0.3 ^a^ D1: 2.8 ± 0.4 ^ab^ D2: 2.4 ± 0.4 ^bc^ D3: 2.1 ± 0.2 ^c^ D4: 2.2 ± 0.5 ^c^	S: 18.8 *** D: 18.3 *** S × D: ns
Moisture	S1: 5.1 ± 0.7 S2: 5.3 ± 0.9 S3: 5.3 ± 1.0	C: 4.2 ± 0.7 ^a^ D1: 4.5 ± 0.4 ^a^ D2: 5.6 ± 0.5 ^b^ D3: 5.6 ± 0.4 ^b^ D4: 6.1 ± 0.2 ^b^	S: ns D: 29.6 *** S × D: ns
Solubility	S1: 5.2 ± 0.7 ^a^ S2: 5.7 ± 0.8 ^b^ S3: 5.7 ± 1.0 ^b^	C: 5.0 ± 0.3 ^a^ D1: 4.5 ± 0.4 ^a^ D2: 5.8 ± 0.6 ^b^ D3: 6.1 ± 0.7 ^bc^ D4: 6.3 ± 0.3 ^c^	S: 11.1 ** D: 44.0 *** S × D: 4.2 **
Creamy	S1: 4.0 ± 0.7 S2: 4.3 ± 1.0 S3: 4.5 ± 1.0	C: 3.3 ± 0.7 ^a^ D1: 3.6 ± 0.4 ^a^ D2: 4.9 ± 0.5 ^b^ D3: 4.5 ± 0.4 ^b^ D4: 5.2 ± 0.7 ^b^	S: ns D: 16.4 *** S × D: ns

Values followed by the same letter within the same column are not significantly different (*p* > 0.05) according to Tukey’s multiple range test, * *p* < 0.05; ** *p* < 0.01; *** *p* < 0.001. ^1^ Season means include the values for the control cheeses, S1: spring; S2: summer; S3: fall. ^2^ Lactase dose (g/L milk): C = control; D1 = 0.125; D2 = 0.25; D3 = 0.5; D4 = 1.0.

## Data Availability

The data presented in this study are available on request from the corresponding author.
